# Development of Skin-On-A-Chip Platforms for Different Utilizations: Factors to Be Considered

**DOI:** 10.3390/mi12030294

**Published:** 2021-03-10

**Authors:** J. Ponmozhi, S. Dhinakaran, Zsófia Varga-Medveczky, Katalin Fónagy, Luca Anna Bors, Kristóf Iván, Franciska Erdő

**Affiliations:** 1 Microfluidics Laboratory, Department of Mechanical Engineering, IPS Academy-Institute of Engineering Science, Indore 452012, India; jponmozhi@gmail.com; 2 The Centre for Fluid Dynamics, Department of Mechanical Engineering, Indian Institute of Technology Indore, Indore 453552, India; sdhina@iiti.ac.in; 3 Faculty of Information Technology and Bionics, Pázmány Péter Catholic University, Práter u. 50a., 1083 Budapest, Hungary; varga-medveczky.zsofia@itk.ppke.hu (Z.V.-M.); fonagy.kata@gmail.com (K.F.); lucaannabors@outlook.com (L.A.B.); ivan.kristof@itk.ppke.hu (K.I.); 4 Heart and Vascular Centre, Faculty of Medicine, Semmelweis University, 1122 Budapest, Hungary

**Keywords:** skin, membranes, reconstructed skins, dermal barrier, shear stress, skin-on-a-chip device, mathematical modelling, CFD, heat transfer, drug diffusion through the skin

## Abstract

There is increasing interest in miniaturized technologies in diagnostics, therapeutic testing, and biomedicinal fundamental research. The same is true for the dermal studies in topical drug development, dermatological disease pathology testing, and cosmetic science. This review aims to collect the recent scientific literature and knowledge about the application of skin-on-a-chip technology in drug diffusion studies, in pharmacological and toxicological experiments, in wound healing, and in fields of cosmetic science (ageing or repair). The basic mathematical models are also presented in the article to predict physical phenomena, such as fluid movement, drug diffusion, and heat transfer taking place across the dermal layers in the chip using Computational Fluid Dynamics techniques. Soon, it can be envisioned that animal studies might be at least in part replaced with skin-on-a-chip technology leading to more reliable results close to study on humans. The new technology is a cost-effective alternative to traditional methods used in research institutes, university labs, and industry. With this article, the authors would like to call attention to a new investigational family of platforms to refresh the researchers’ theranostics and preclinical, experimental toolbox.

## 1. Introduction

**Figure 1:**
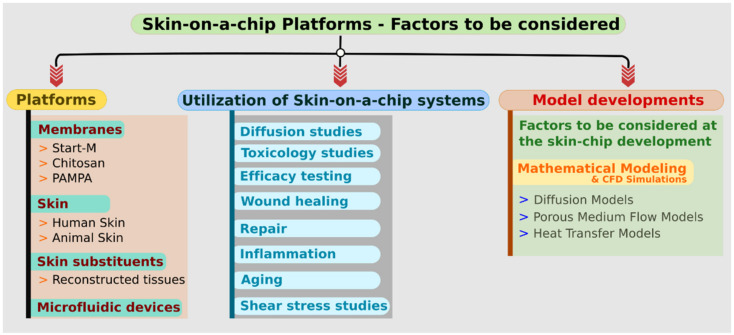
Chart showing the different factors to be considered in the case of skin-on-a-chip platforms.

During the last decade, there was an increasing number of reports published about the developments of microfluidic platforms for testing various organs (liver, kidney, heart etc.), including the skin in microscale, characterizing its properties and behavior in certain physiological and pathological conditions. These studies aimed to replace the animal experiments, and with micronization, they could reduce the costs of experimental setups and the use of tissues or tissue substituents. The revolutionary approach of microfluidic devices is under introduction in different fields of research and industry. In the current review, the authors focused on skin-on-a-chip technology and the use of these devices in different areas of dermal investigations. First, a general introduction is given to the skin-on-a-chip platforms, including the diffusion surface materials (membranes—STRAT-M^®^, chitosan, parallel artificial membrane permeability assay (PAMPA)—skins with animal and human origin, and also the skin equivalents including epidermal and full-thickness skin models). Then the differentially engineered microfluidic platforms are presented based on the recent literature. In the third part of the article, the possible utilizations of the skin-on-a-chip platforms are listed. These applications include drug diffusion studies, toxicological studies, efficacy testing, ageing, repair, inflammation, wound healing, and shear stress studies. The next part of the paper focuses on the factors to be considered during the development of such microfluidic devices. Mathematical modelling, diffusion models, fluid flow models, and heat transfer models for the skins are described. As the topic is relatively new, the number of scientific references is still limited. In this review, we provide a summary to help the readers collect ideas for the design, fabrication, and utilization of similar microfluidic equipment for their purposes. Figure 1 provides a brief overview of the topics presented in this review.

## 2. About the Platforms

**Figure 2:**
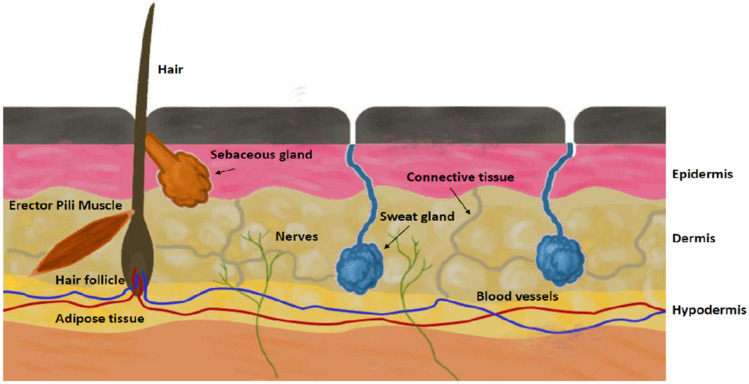
The anatomical structure of human skin.

Artificial membranes reconstructed skins or excised skin samples find application in in-vitro and ex-vivo dermatological, pharmaceutical, and cosmetological studies. Depending on the research and development phase and the fundamental question of the study (permeability, irritation, corrosion, toxicity, disease models, pharmacology, therapeutic approaches, pharmacokinetics, and formulation optimization), different preparations are the optimal platforms for testing. The structure of the human skin mimicked with different models is shown in Figure 2.

### 2.1. Membranes

In the complexity order of the testing platforms, artificial membranes are the simplest elements. The artificial membranes are fabricated to substitute human or animal skin samples and can be easily purchased and stored before the experiments [1]. A summary of the advantages and shortcomings of different membranes used as skin analogous are presented in Table 1. These membranes have the following standard features: (1) They contain pores; (2) are chemically inert; (3) have high compatibility with solvents; and (4) are commercially available. Many investigators employ them as a model membrane in diffusion cell experiments for topical product characterization purposes. However, these membranes are also intrinsically different in that they all possess diverse thickness, porosity, tortuosity, and polymer materials, which many researchers have overlooked. The permeability results gained with these membranes are well-reproducible, with relatively low variability [2]. A further advantage is the lack of ethical concerns contrary to human or animal tissue. The majority of the scientific reports describe silicon-based membranes such as Silastic, Polydimethylsiloxane (PDMS), and Silatos [3]. The traditional cellulose-acetate membranes are also frequently used, mainly in the first phase of the diffusion studies [4]. Strat-M^®^ (Merck Millipore) is a recent development in the field of synthetic membranes, and it consists of multiple layers of polyethersulfone. The following factors should be considered when selecting a membrane for topical drug diffusion studies: (1) The high flux membranes for formulation analysis should have a higher than 60% porosity, tortuosity of 1, and relatively thin (~10 μm); (2) synthetic membranes for microfiltration are preferred for diffusion cells studies; and (3) membranes with coatings are not favorable [5]. In the last several years, chitosan-based and Strat-M^®^ synthetic membranes have become quite popular in various research fields [6,7,8].

#### 2.1.1. Strat-M^®^

**Figure 3:**
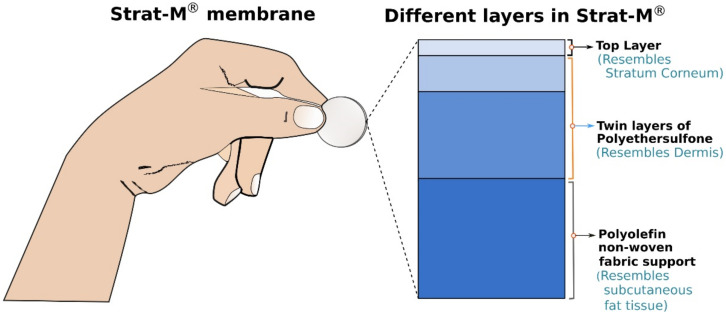
A Strat-M^®^ membrane and the representation of different layers.

The most physical barrier for permeation across the human skin is the dead corneocyte layer of the stratum corneum, the outermost surface of the epidermis and tortuous lipid pathway as well. The rate-limiting layer of corneocytes is not as characteristic in artificial membranes as in the skin. The Strat-M^®^ membrane has multiple layers (See Figure 3), and they have different permeability. The upper layer consists of two sublayers of polyethersulfone (PES, more resistant to diffusion), while the lower layer includes polyolefin, which is a more permeable layer. Strat-M^®^ membrane can predict both lipophilic and hydrophilic molecules’ absorption [1,10]. In general, the Strat-M^®^ membranes are more permeable for hydrophilic than for lipophilic drugs. For screening purposes, Strat-M^®^ seems to be a cost-effective option contrary to excised skins in testing topical drug and cosmetic formulations and active ingredients in the different diffusion studies. In the majority of studies, Strat-M^®^ membrane shows a good correlation in permeability with the skin tissues.

#### 2.1.2. Chitosan

Chitosan, a polysaccharide, can be produced in different chain sizes from fungal (white mushrooms) and animal (shrimp shells) sources. Dense and porous chitosan-alginate membranes can be used as a dressing for skin wounds treated cell therapy. By adding Poloxamer 188 to the formulations, one can generate thick, porous membranes. Bierhalz and coworkers evaluated the influence of chitosan types on membranes’ physicochemical properties and toxicity to fibroblasts [11]. Porosity was higher in membranes produced with fungal chitosan and increased with its molecular weight. These fungal high molecular weight membranes showed the most increased thickness, roughness, opacity, liquid uptake, and water vapor permeability in the study. The membranes were not toxic to fibroblasts, but researchers obtained the lowest cytotoxicity values for membranes with fungal chitosan treated with surfactant. Fungal chitosan membranes benefit from replacing chitosan from animal sources due to low cytotoxicity and good physicochemical properties [11].

#### 2.1.3. PAMPA

**Figure 4:**
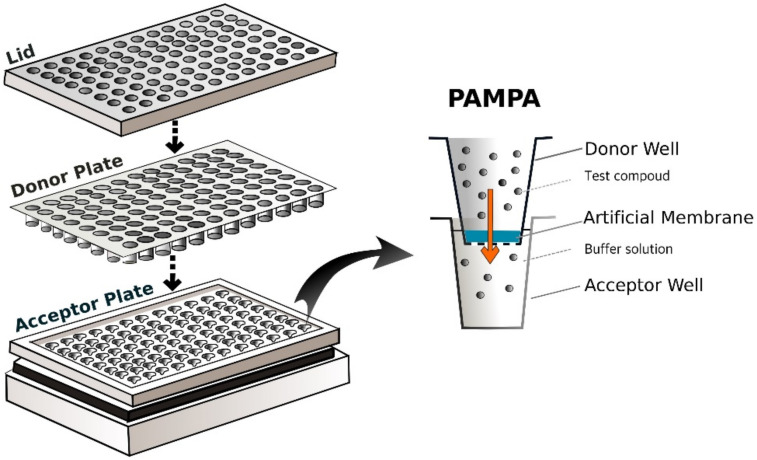
Parallel Artificial Membrane Permeability Assay (PAMPA) 96-well setup (left); a single well of PAMPA (right).

Other approaches to gain preliminary permeability data in-vitro include the artificial membrane-based PAMPA (Parallel Artificial Membrane Permeability Assay) systems. The first commercially available PAMPA for performing penetration studies was the skin-PAMPA, supplied by Pion Inc. In PAMPAs, an artificial membrane (the membrane itself is composed of free fatty acids, cholesterol, and a synthetic ceramide analogue) separates the donor and acceptor compartments of the cells in the 96-well plate arrangement, as shown in Figure 4. The donor compartment contains the test compound, while in the acceptor compartment, a buffer solution is placed. The PAMPA systems are famous for fast high-throughput screening of compounds to determine their permeability profile. The results of different research groups showed a good correlation measured using PAMPA diffusion cells with the results of other groups running experiments in silicone membranes [12,13,14,15].

Although synthetic membranes have many advantages over human/animal skins, they cannot match the barrier function of the human stratum corneum and epidermis [1,16].

### 2.2. Skins

There are several advantages when ex-vivo models of human or animal skins are employed instead of in-vivo methods. These include (1) precise, controlled conditions and more ease in performing experiments; (2) allows the use of dangerous or toxic chemicals; (3) several parallel experiments can be run simultaneously; (4) human tissues from different sources can be used, which can provide predictive data; (5) cosmetics can also be tested without any ethical problems [17], (6) different formulations can be compared precisely; and (7) pharmacokinetic parameters can be directly studied.

#### 2.2.1. Human Skins

Excised human skin is usually obtained from plastic surgeries, either from hospitals or from tissue banks. It is primarily used to assess transdermal penetration, necessary for efficacy and safety evaluation of chemicals, plant protection products, pharmaceuticals, and cosmetics [1,18]. Ex vivo skins can be prepared in various thicknesses using a dermatome. It is available as epidermis and a portion of dermis (~100–400 μm) or full-thickness skin samples containing epidermis and dermis (depending on anatomical location up to 1–2 mm). Variations in the sample thickness impact the drug penetration to the receptor compartment [19]. Several authorities [20,21,22] prefer the well-defined thickness skin preparations for reproducible testing of the drug permeability.

#### 2.2.2. Animal Skins

As an animal skin model, the pig skin is described as the best comparable model of the human skin, both structurally and the barrier function [23,24] (Table 2). The excellent availability is also an essential point of the use of excised porcine skins. Rat skin is also widely used for dermal penetration studies [24,25,26]. Rat skin is employed in the plant protection field for toxicology studies and in-vivo skin absorption studies (transdermal microdialysis). However, it is known that the permeability of rat skin is much higher than that of the human skin [27] (higher density of hair follicles, higher transappendageal absorption routes, lower thickness), and also the metabolism is very different from the human. Therefore, the results obtained with rat skins should be interpreted with care [28,29,30]. The human ex vivo and rat ex vivo and in vivo data are currently accepted for different aspects of risk assessment in the EU and NAFTA (North American Free Trade Agreement) countries [31].

### 2.3. Skin Substituents

#### Reconstructed Tissues

The development of different skin substituents started almost three decades ago. First, the skin models aimed to mimic skin, and for this purpose, the normal human keratinocytes (NHKs) were seeded on the dermis [33]. Later the model was developed, and the NHKs were grown on supporting membranes and formed reconstructed human epidermis (RHE) [34]. We can talk about two main skin models: RHE models and the full-thickness human skin model (living skin equivalents (LSEs)). To produce RHE, NHKs are proliferated to the multilayered epidermis. The full-thickness LSE model includes both epidermis and dermis layers. Commercially available RHEs are summarized in the table below (Table 3).

The advantages and limitations of 3D tissue-engineered skin models are summarized in Table 4.

Keratinocytes make up 95% of the cells in the epidermis and play an integral role in initiating, modulating, and regulating skin irritation [54]. Therefore, normal human epidermal keratinocytes can be cultured to form a multilayer, differentiated model and seeded on matrices of either dermal components or non-biological scaffolds [55,56,57,58].

### 2.4. Microfluidic Devices

**Figure 5:**
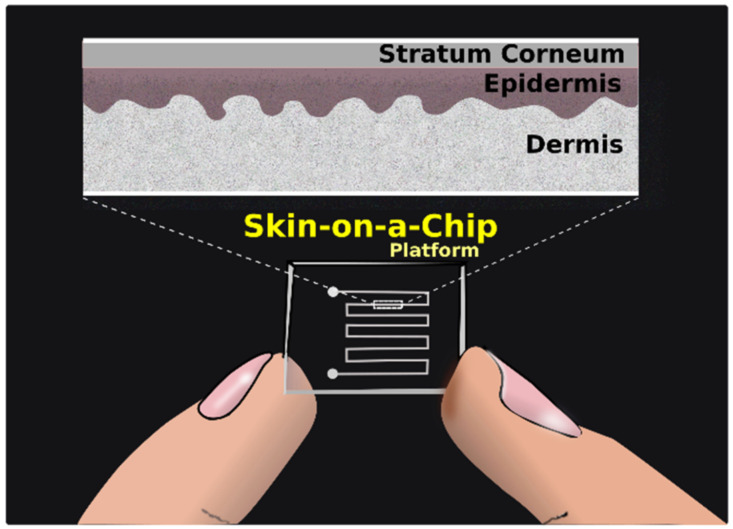
Sketch of a microfluidic skin-on-a-chip platform.

Microfluidics technology can precisely control the fluidic components by flow rate settings, physiological composition adjustment, and ensure the communication between different tissues or cell constructs and the fluids at a micro-scale. These platforms are cost-effective, for example, for drug screening purposes. Abaci and co-workers transferred skin constructs onto a microfluidic platform that allows for the long-term maintenance of the artificial tissue at physiologically relevant nutrient supply rates [59]. Kim and co-workers built up a device where human skin equivalent (HSE) included epidermis and dermis, and they were cultured in skin-on-a-chip (similarly to the Abaci groups system, they used a pumpless chip where the gravity provided the continuous flow rate. These skin-on-a-chip platforms (see Figure 5) could re-circulate the medium at reasonable flow rates without a pump. It was demonstrated in this system that the anticancer drug, doxorubicin, had direct toxic effects on keratinocyte proliferation and differentiation [59]. Other studies focused on co-culture of skin tissue biopsies with other organs, such as liver, intestine, and kidney, on a microfluidic platform in a transwell format [60,61,62]. Several proof-of-concept studies demonstrated the long-term maintenance, function, and response of these biopsy cultures to drug toxicity. The multiple organ studies using tissue biopsies are very important for giving significant insights for the maintenance of multiple engineered tissues on a single platform.

The microfluidic platform designed, fabricated, and validated by Alberti and co-workers offers an excellent alternative to traditional Franz diffusion cells that are costly, low-throughput, and not always well-reproducible [63]. Microfluidics has the potential to overcome these drawbacks, and this research group proved this by using three model chemicals of varying lipophilicity: Caffeine, salicylic acid, and testosterone.

Our research group constructed a similar platform [24,26]. In this microfluidic chip, human and animal excised skin samples were used in permeability testing. Our laboratory validated the system also functionally for testing transdermal absorption of topically applied P-glycoprotein substrates (quinidine and erythromycin). The effect of freezing and thawing on the efflux transporter function at the epidermis and dermis border zone was also successfully evaluated in the microchip [24].

**Figure 6:**
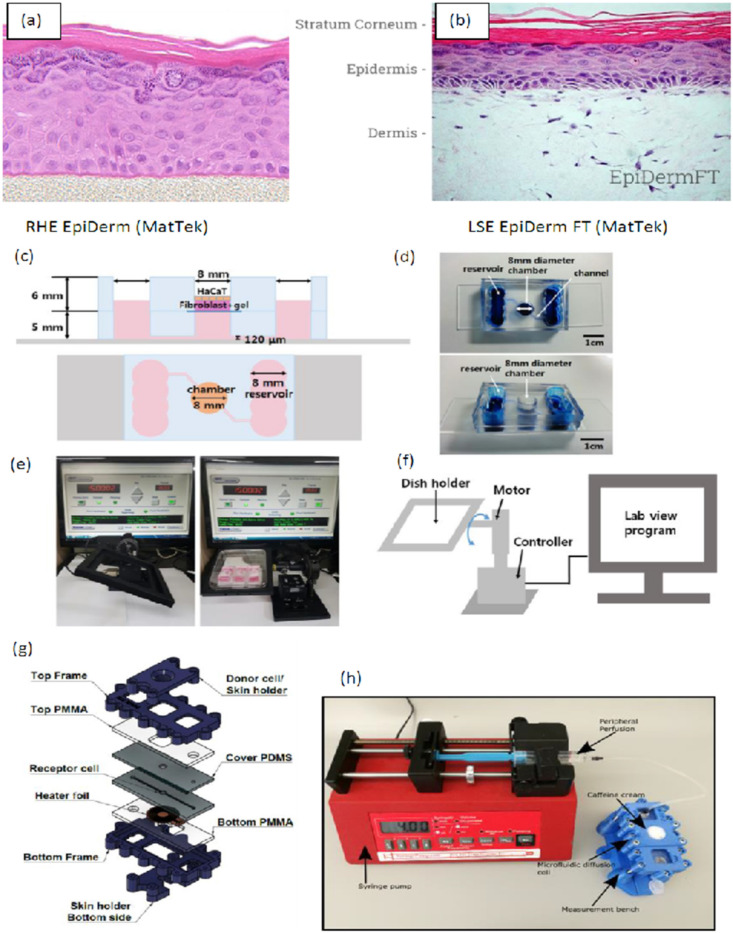
(**a**) Human reconstructed epidermis model of MatTek, (**b**) human full thickness reconstructed skin model by MatTek, (**c**) cross-sectional and top schematics of the skin chip by Lee and co-workers with permission, (**d**) pictures of the assembled skin chip, (**e**) picture of the gravity-flow control system, (**f**) schematics of the gravity-flow control system [64]. (**g**) Structure of skin-on-a-chip designed by Lukács et al. 2019 [26], (**h**) diffusion measurement setup by Bajza et al. 2020 [24].

Lee and co-workers aimed to develop a microfluidic, three-dimensional (3D) skin chip with fluidic channels using PDMS and hydrogels. Mass transport within the collagen hydrogel matrix was verified with fluorescent model molecules, and a transport-reaction model of oxygen and glucose inside the skin chip was developed to aid the design of the microfluidic device. The viabilities of dermal fibroblasts and HaCaT (spontaneously transformed aneuploid immortal keratinocyte cell line from adult human skin) culture were compared in the chip with various culture conditions. The results suggested that the presence of flow plays a crucial role in maintaining viability. This 3D skin chip with vascular structures can be a valuable in vitro model for reproducing the interaction between different components of the skin tissue and works as a more physiologically realistic platform for testing skin reaction to cosmetic products and drugs [64]. The skin models and some chip structures are shown in Figure 6.

A comparison of traditional diffusion cell systems and skin-on-a-chip devices is shown in Table 5.

The different fabrication technologies, the materials, and the fields of application of skin-on-a-chip systems are summarized in Table 6.

## 3. Utilization of Skin-On-A-Chip Systems

The use of artificial membranes reconstructed human skins, ex vivo excised skin preparations, or in vivo skin models might be different. Here we summarize the most important application areas of these skin-on-a-chip test systems in the field of dermal fundamental research and pharmaceutical or toxicological applied research studies.

### 3.1. Diffusion Studies

The drug penetration through the dermal barrier is an essential factor regarding the topical and systemic effects expressed by the active ingredients of pharmaceutical or cosmetic compositions. It is crucial to know the absorption process of the topical compounds for assessment of pharmacokinetics, pharmacological and toxicological profiles. Skin penetration can be studied in different in vitro and ex vivo systems. This review is focusing on the examinations in skin-on-a-chip devices. Lukacs and co-workers described the validation of a skin-on-a-chip microfluidic device by caffeine cream and excised animal skin preparations for applicability in dermal diffusion testing [26]. This project was continued by Bajza and co-workers, who investigated the efflux transporter function in the skin using skin-chip devices. Two P-glycoprotein model substrates (erythromycin and quinidine) were tested in cream and gel formulation in an ex vivo skin-on-a-chip instrument. The device’s parallelization can also be implemented, as demonstrated in the study [24]. The findings indicate that the skin-chip system is an appropriate solution for the screening of skin penetration and drug-drug interactions in the dermal barrier.

Abaci and co-workers [59] demonstrated that their mathematical model can be used to estimate intrinsic skin transport properties such as diffusion rate and drug partitioning, independent of skin thickness. Besides, HSE in a microfluidic model maintained skin barrier functions for three weeks and was a cost-effective in-vitro platform for drug testing.

### 3.2. Toxicology Studies

Several in vitro dermal toxicity models are available for the assessment of potential local toxicity of test compounds. These methods are suitable for testing sensitization, skin irritation, corrosion, and skin phototoxic effects, and they are continually being developed, and the assays are validated for regulatory purposes [79,80]. In the review of Chong and co-workers, it was demonstrated that the microfluidic chip models could be used to detect drug toxicity. Several biomarkers of toxicity were determined by organ-on-a-chip technology [81].

Sriram and co-workers developed a full-thickness skin-on-a-chip culture model and in vitro assay protocol. Their microfluidic chip contained independent tissue culture (fibroblasts with N/TERT keratinocytes of humans) and analysis units. They demonstrated that the innovative microfluidic design combined with state-of-the-art epithelial tissue culturing significantly improved the quality and functionality of the device [73]. Microfluidic chip models of kidney, heart, nerve, liver, and other organs highlighted the importance of applying these models for general drug toxicity detection.

Skin irritation is physiologically induced by vasodilation and increased permeability of dermal microvascular endothelial cells. Jeon and co-workers attempted to mimic physiological skin irritation using a skin-on-a-chip model and compared predictive capacities with a reconstructed human epidermis to evaluate its effectiveness. The skin-on-a-chip model, consisting of three skin layers, the epidermal, dermal, and endothelial components, was adapted. The research group provided evidence that the dual-parameter chip model possesses enhanced predictive capacity and could serve as an alternative to animal testing for skin irritation [82]. In chemically induced skin irritation, the test substance passes through the skin and causes increased permeability of endothelial cells, vasodilation, and edema. The tight junctions (TJ) can be observed in the microvascular elements of the dermis and measured to assess physiological responses to the drugs/chemicals. When the TJs dissociated by test substances, cellular permeability increases. Tavares and co-workers assessed the tissue viability according to OECD (Organisation for Economic Co-operation and Development) test guideline no. 439 as well as changes in homeostasis (EGFR, HSPB1) and metabolism (NAT1) and also inflammation (IL-1, IL-6, IL-8), after topical fucoxanthin treatment. They tested the suitability of a 24-well-based reconstructed human skin device for testing irritation [83].

### 3.3. Efficacy Testing

The protective function of the skin is impairing during skin reactions such as inflammation, irritation, allergies, or malignancies. Skin-on-a-chip systems can be used to model human skin diseases. In the study of Wufuer et al., it was demonstrated that the applied skin model successfully mimicked skin inflammation and edema. The model can be used in drug testing to measure the efficacy of the therapeutic drug (e.g., dexamethasone) on reducing tumor necrosis factor-alpha (TNF-α)-induced inflammation and edema [71].

Mori and co-workers fabricated a 3D skin-on-a-chip microdevice with vascular channels coated with endothelial cells that comprised a skin equivalent fixed to a culture device connected to an external pump and tubes. The model can be used for the development of skin therapies and cosmetics [77]. Although the skin-on-a-chip model is vital for determining the root causes of skin diseases and effective treatment for clinical applications, some challenges remained, like the skin microenvironment and its heterogeneous structure (hair follicles, sebaceous glands, sweat glands, nerves, and vascular elements) [84].

### 3.4. Wound Healing

Skin is to act as physical barriers that protect the tissues from chemical, physical, and microbial agents. Early in vitro skin models as reconstructed human epidermis (RHE) models consisted of two dimensional and later developed three-dimensional cell cultures [85]. RHE models can be used to study permeability and absorption; however, they do not include endothelial cells and evaluate irritation based on cell viability (CV) [82]. During wound healing, angiogenesis plays a major role that brings oxygen and nutrients to the growing tissues and removes catabolic wastes. In this way, angiogenesis assists the repairing of post-burn wound tissues. Therefore, to mimic wound pathology and test therapeutics, only vascularized skin substituents can be applied to improve wound healing.

The microenvironment and physiological responses play key roles in acute and chronic wounds. In the complex wound healing process, the most critical phases are hemostasis, inflammation, and cell proliferation. The Skin-on-a-chip model can be an in vitro alternative to in vivo systems for studying cell migration in the wound healing process and the effect of therapeutic interventions. Biglari and co-workers developed a microfluidic wound-on-chip model to mimic the inflammatory phase and provide more information about the behavior of different cell types involved in wound healing [86]. The microfluidic wound-on-chip device was used in this study for the high-throughput screening of anti-inflammatory compounds.

### 3.5. Repair

The human body’s outermost barrier is the skin, which has a vital role in protecting and demarcating the body from the environment. When the skin is damaged, it activates cytokines’ expression, causing inflammation in the damaged area and fighting against the potential intruder pathogens. Angiogenesis and the pro-angiogenic factors like PDGF (platelet-derived growth factor), VEGF (vascular endothelial growth factor), and TGF-β (transforming growth factor β) can help in the regeneration of the tissue by stimulating the proliferation, migration, and tube formation of endothelial cells [87,88,89,90]. Studies have shown similarities in the skin defense reaction and repairing mechanism in high UV exposure and skin surface damage, including inflammation, increased cell migration, and proliferation [83,91]. Shortcomings of traditional tissue engineering have driven the fast development of vascularized skin tissue production, leading to new technologies such as 3D bioprinting, nano-fabrication, and micro-patterning using a hydrogel-based scaffold. The key hope to bioprinting would be the generation of interconnected functional vessels, coupled with the addition of specific cell types to mimic the biological and architectural complexity of the native skin. Furthermore, stem cells have been gaining interest due to their highly regenerative potential and participation in wound healing. Many bioprinting [92,93,94,95,96] and skin-on-a-chip methods have been presented as promising alternatives replacing animal experiments to study these processes.

### 3.6. Inflammation

Like any other part of the body, the skin can be involved in immune responses. Inflammation in the skin often causes a rash to form. It is typically a response from immune system to conditions such as infections, internal disease condition, or allergic reaction. Some of the symptoms of skin inflammation can include rash, which may vary depending on the cause of the inflammation. It can be smooth or scaly, may itch, burn, or sting, and may be flat or raised. The inflammation in the skin can cause redness, warmth in the affected area, blisters or pimples, raw or cracked areas of skin that may bleed, and thickening of skin in the affected area.

As different warning signs appear in a tissue, cytokine expression and release increases for activating the defense of the body. This signal cascade can be started by tissue damage, pathogens, and foreign bodies, but in the case of the skin, it can also be the consequence of high exposure to UV light [83,88,89,90].

The irritation and inflammation tests of topical drugs and cosmetics is a very controversial topic in animal research. Without testing, these substances can be a risk of toxicity and allergic reaction to the human skin; therefore, the testing should not be cancelled. However, alternative methods can replace the use of animals. One of these alternatives are skin-on-a-chip methods [71,97]. Tavares et al. developed an organ-on-a-chip method built up with reconstructed human skin culture (consisting of keratinocytes and fibroblasts) to test the topical irritation caused by the application of fucoxanthin [83]. Fucoxanthin proved its antioxidant and cytoprotective functions, but the topical safety has not been characterized earlier in human cell-based models. Changes in inflammation (IL-1α, IL-6, IL-8), homeostasis (EGFR, HSPB1), and metabolism (NAT1) markers were monitored in this study [83].

### 3.7. Aging

When cellular senescence develops, the skin is exposed to harmful environmental risks that speed up aging. Several organ-on-a-chip methods were developed to study skin aging.

In the papers of Kim et al. a pumpless skin-on-a-chip device was used as a skin model to investigate the anti-aging effect of curcumin and coenzyme Q10 [89,98]. The skin culture consisted of rat tail collagen, human dermal fibroblast, and epidermal keratinocytes [98]. The chip was made of polydimethylsiloxane (PDMS) with an 8 mm cylindrical chamber in the middle and two chambers for the medium on the sides, which were connected through a lower channel. A porous membrane was placed between the cell culture skin substituent and the channel. This model was used for investigating the anti-aging and antioxidant effects of coenzyme Q10 in the skin. Another experiment used gamma-ray radiation on a cell culture made from primary human umbilical vein endothelial cells and primary human skin fibroblasts HCA2 to model cellular senescence in the skin [99]. The aged phenotype was checked with the canonical β-galactosidase assay. PDMS-based microchips were used to examine a 3D model of microvessels with young or senescent skin fibroblasts in its surrounding. It was found that senescent fibroblasts mechanically altered the extracellular matrix and induced sprouting angiogenesis of the microvessels via their senescence-associated secretory phenotype. The authors concluded that mechanical changes of the microenvironment play an important role in sustaining senescence-associated secretory phenotype-induced angiogenesis as it was observed in the microchip.

### 3.8. Shear Stress Studies

Shear stress is caused by friction between fluid particles. In skin-on-chip, shear stress can be defined as the frictional force of fluid (biofluid/chemical/reagents/nutrients) that acts tangentially on skin cells/tissues. Shear stress is influenced by the viscosity, velocity, and temperature of the fluid.

Shear stress influences the arrangement, alignment, growth, and dense population of the skin cells in the microfluidic chip with its low, reciprocating, and high shear stress. Table 7 explains the effect of shear stress on the skin cells in a few application areas.

Artificial microfluidic skin with two layers to visualize the perspiration associated with human sweating is developed to experiment with any wearable material on the skin (material, device, or product) by Hou and his group. The lower layer mimics the sweat gland’s secretory portion by providing a constant sweat flow rate with a pressure drop through a 2 µm gap. The top layer is fabricated with a material that can mimic the skin wetting surface with the required pore density and pore diameter of 50 µm [107].

**Figure 7:**
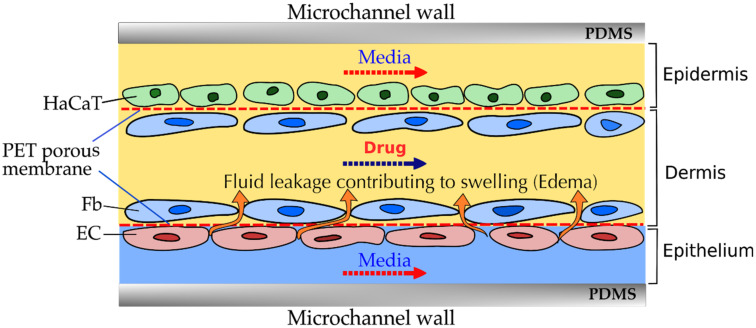
The cross-sectional view in a microchannel with three layers of skin cells with different culture media flowing at different flow rates to visualize inflammation, edema, and drug-based treatment.

To overcome the limitations of the collagen-based skin equivalents, an enhanced skin equivalent is made with different layers separated by membranes that have 1 µm pore size and a pore density of 2 × 10^6^ pores/cm^2^. The fibrin-based dermal equivalent (DE) was cultured in one of the lower compartments at a flow rate of 1.0 µL/min. Keratinocytes were seeded over the DE and submerged in the serum-free medium for culturing. These skin equivalents are prepared under continuous supply and drainage of nutrients and metabolites [73]. Lee and his group prepared endothelium with dermis and epidermis in order to mimic the inflammation and edema. PET porous membranes were included between each skin layer to allow the transfer of drugs, cytokines, and nutrients (Figure 7). A diverse culture medium was provided to the three different skin layers at different flow rates. They reported the permeability of the cells with and without medicine by linking inflammation with paracellular spaces. They used this developed model to screen drugs and replace animals studies [71].

The same group worked on a similar platform for simulating the skin irritation with the HaCaT cells to mimic the epidermal component and was used in the top layer; the Hs27 to mimic the dermal component and placed in the middle layer; HUVECs to mimic the endothelial component in the bottom layer. They have reported that this developed model was better in terms of sensitivity, specificity, and accuracy when compared with other models to differentiate the skin irritants and non-irritant [82].

Yung-Shin Sun group demonstrated that wound healing increased with higher shear stress. The authors seeded a microchannel with mouse embryonic fibroblast cell line NIH/3T3. They prepared wounds over this layer by passing trypsin at 400 µL/min and culture medium at 800 µL/min and analyzed the healing rate with ß-Lapochone at different shear stress and concentration on the varying wound area. They reported that the healing rate was more for higher shear stress of the medicine for a larger wound area at a concentration of ß-Lapochone—0.5 µM [102]. Human skin keratinocyte was cultured in a microchannel to show that the skin cells are responsive to the shear stress of the fluid flowing over it, during embryogenesis, as the maturation of skin develops under the influence of amniotic fluid. They showed through their experiments that low shear stress of 0.06 dyne/cm^2^ has reorganization in keratinocyte cells, and high shear stress of 6 dyne/cm^2^ had cellular disruption [108].

Towards developing a native skin, a research group from Germany cultured a complete hair follicular unit in 14 days. During the culture period, they observed hair-shaft elongation in growing anagen and decreasing catagen of hair follicles. The follicular hair unit extracts from males undergoing hair transplantation surgery were submerged in the medium stream with a flow rate ranging between 7–70 µL/min. This flow rate in the chip would affect many factors, including cell–cell communications, extracellular gradients, and local concentrations of secreted ligands of tissues maintained in the microchannel [60]. Further hair follicle growth to study the penetration of hair dyes, chemicals (perfumes, house cleaning products, and agricultural products) through the hair shaft and the regrowth of hair follicles near the wound repaired area is further studied [109].

## 4. Model Developments

### 4.1. Factors to Be Considered at the Skin-Chip Development

For performing drug toxicity experimental studies with microfluidic skin-on-a-chip platforms, skin cells are grown within a microchannel as shown in Figure 7. Drugs as well the media are allowed to pass through the channel in the presence of cell layers. Drug diffusion and fluid leakage occurs across the cell layers that have a definite porosity and permeability. Depending on the values of these parameters, fluid leakage and drug diffusion may or may not occur. Computational Fluid Dynamics techniques can be used to predict the flow as well as scalar transport in such devices beforehand. With the predicted flow and concentration field, the porosity and permeability of the skin layers can be varied accordingly. This can greatly reduce effort and time spent in performing multiple experiments.

Human (or animal) skin and artificial membranes (such as Strat-M^®^) are composed of different layers of cells and artificial materials, respectively. These layers are porous, with each layer having a definite porosity and permeability. As a whole, the entire skin or artificial membrane can be considered a composite porous medium. In a porous medium, factors such as permeability (k), porosity (ϵ), diffusion coefficient (D) and fluid velocity (*v*), pressure (P); specific heat (C_p_), among others, play a prominent role in drug diffusion, fluid flow and heat transfer. In a microfluidic platform, permeability dramatically affects the pressure field (when fluid flows) and shear stress. Here we give a brief introduction to mathematical modelling and CFD simulations and the basic mathematical models that can be utilized for carrying out a study on the fluid flow, heat, and mass transfer in porous media. The models described below, which are simple and do not involve too many parameters, can be adopted for studies dealing with microfluidic skin-on-a-chip platforms where fluid flow, drug diffusion, and heat transfer occurs.

#### 4.1.1. Mathematical Modelling and CFD Simulations

Computational Fluid Dynamics (CFD) uses mathematical techniques to provide a quantitative prediction of fluid flow, heat, or mass transfer phenomena based on conservation laws [110]. It can be applied in studies involving such phenomena in the human skin, artificial membranes, and microfluidic skin-on-a-chip devices. Physical phenomena such as fluid percolation, heat, and mass transfer across the dermal layers can be represented by mathematical models (i.e., mathematical equations) and solved using appropriate CFD tools. Precisely, the technique consists of the following steps: (1) Defining the geometry of the skin layers or the devices containing the tissues/artificial membranes; (2) meshing the geometry; (3) choosing the appropriate equation governing the physical phenomena; (4) solving the governing equations with relevant numerical methods; (5) and post-processing and analyzing the obtained numerical data [111]. CFD technique has been successfully applied in transdermal drug delivery studies involving microfluidic/skin-on-a-chip platforms [112].

#### 4.1.2. Diffusion Model for Dermal Layers

**Figure 8:**
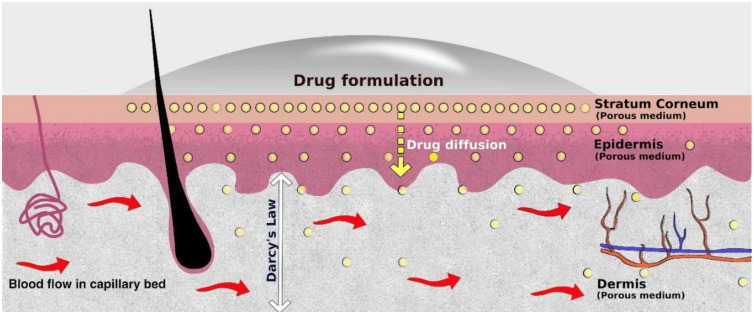
Representation of different layers of the skin along with the diffusion of drugs in the skin. The layers can be considered as a porous medium and the porous medium equations can be written for the mass transfer across the SC, Epidermis, and Dermis. For the blood flow in the tissues, the Darcy model can be adopted.

The skins consist of four primary layers, namely the stratum corneum, epidermis, dermis, and hypodermis. When a drug formulation is applied over a skin surface, they penetrate the skin through hair follicles and sebaceous glands [113]. They also penetrate across the stratum corneum by molecular diffusion, as shown in Figure 8. The stratum corneum is a heterogeneous material composed of corneocytes and lipid matrix. The physical organization of the SC (i.e., the porosity) determines its barrier property. The alterations in the formation of the SC gives rise to varied porosity as well as permeability. In the SC, the changes in lipids composition, temperature, water contents, pH, etc., affect the arrangement of solid and fluid lipid domains that govern the variation in SC permeability. The dermis, which is the layer next to the epidermis, contains blood vessels, glands, follicles, and other structures. After passing through the SC and other layers of the epidermis, the applied drug formulations reach the dermis, where it is absorbed into the capillaries and sent to the circulatory system. The SC, epidermis, and dermis constitute a dynamic porous medium [114]. Therefore, porous medium models can predict drug diffusion and fluid movement in these layers.

The following equations governing the diffusion of drugs through the skin layers given by Narasimhan and Joseph [115] are more suitable for drug diffusion studies in microfluidic skin-on-a-chip devices as they takes into account the unsteady diffusion as well as the porosity of the cell layers. This simple model does not involve too many parameters and can always be used by the experimental community without much loss in accuracy.

**Stratum Corneum:** The equation governing the diffusion of drug formulation through the porous stratum corneum is given by

(1) $$\varepsilon \frac{\partial C}{\partial t}\;+\;\nabla \left(Cv\right)=\;\nabla \;\cdot \left(D_{i}\nabla C\right)$$

where *ε* is the porosity of the dermal layers; *C* is the concentration of the drug; t is the time; *v* is the velocity; *D* is the diffusivity. Here, the effective diffusivity $D=\epsilon \tau D_{F}$.

**Epidermis:** The equation governing diffusion in the porous epidermis is given as

(2) $$\varepsilon \frac{\partial C}{\partial t}\;=\;\nabla \;\cdot \left(D_{i}\nabla C\right)$$

**Dermis:** The dermis is a fibrous structure consisting of collagen, elastic tissue, nerve ends, hair follicles, sweat glands, and vasculature. It can also be considered as a porous medium, and accordingly, the equation governing the drug diffusion in the porous dermis can be written as

(3) $$\varepsilon \frac{\partial C}{\partial t}\;+\;\nabla \left(Cv\right)=\;\nabla \;\cdot \left(D_{i}\nabla C\right)$$

Drug diffusion in the skin layers (or artificial membranes) depends on the following factors: (a) Diffusion coefficient of the drug; (b) porosity of layers/membranes; (c) time period, and (d) the fluid velocity.

#### 4.1.3. Fluid Flow Model for the Dermis

##### Darcy Model

Darcy’s model gives the transport model applicable for flow in the dermis. This model is widely used for calculating flow in a porous medium at small flow velocities. It is presented as [116]

(4) $$u=-\frac{K}{\mu }\nabla P$$

Here, *u* is the macroscopic velocity of blood in the dermis; *K* is the permeability of the dermis; μ is the viscosity of the blood; and ∇P  is the pressure gradient. Along with the Darcy model, the mass conservation equation also needs to be taken into account while performing any simulations. The mass conservation in the porous dermis is given as

(5) $$\nabla \cdot \left(\rho u\right)\;=0$$

SC can be considered a porous medium since corneocytes are impermeable to fluid percolation (water), while lipids give way for water transport [114]. With this assumption, Marquez-Logo and co-workers [117] numerically simulated the water transport through the SC. They used Darcy’s model to model the flow. The authors also simulated mass transport through the SC, considering advection, diffusion, and dispersion. Simulations were performed with groundwater flow and chemical transport modelling software called MODFLOW and MT3D. Narasimhan and Joseph [115], using CFD, numerically investigated the transdermal drug delivery across human skin, considering it to be a composite porous medium. Using commercial software—COMSOL—they analyzed the effect of cell migration and anisotropic diffusivity of stratum corneum on drug diffusion. They solved the unsteady diffusion equation in the SC, epidermis, and dermis modified to account for the porosity of the skin layers. In the dermis, Darcy’s model was solved to simulate the blood flow. The authors observed that cell migration in the SC impedes the motion of drugs into the skin. When SC is assumed to be anisotropic, the drug diffusion was found to be double that of the isotropic case.

In the Dermis, both advection and diffusion of the drug takes place. As seen in Equations (1) and (3), in the case of unsteady drug diffusion, the concentration C depends on the fluid velocity (*u*). The Darcy model accurately predicts the velocity field. Fluid flow in the dermal layers or artificial membranes depends on the following factors: (1) Fluid viscosity, (2) permeability; (3) porosity; and (4) the pressure gradient, among other factors. The higher the permeability, the more ease with which the fluid flows. In a skin-on-a-chip device, Darcy’s model can be used to predict fluid velocities.

##### Darcy-Brinkman-Forchheimer Model

Darcy’s law is linear in nature and it neglects the viscous effects near boundaries. So, extensions to Darcy’s law have been applied in the past, including Brinkman’s equation and Forchheimer equation [118,119]. The Brinkman equation incorporates viscous effects, and the Forchheimer equation incorporates nonlinear drag effects at higher fluid velocities. The Darcy–Brinkman–Forchheimer (DBF) model can be used for modelling flowing and predicting the fluid velocities in a porous medium consisting of fluid as well as porous layers (cell layers) such as the microfluidic skin-on-a-chip geometry shown in Figure 7. The DBF model would be particularly useful when the velocity of the media or the drug formulation is high in the microfluidic skin-on-a-chip devices. It is given by [120,121]

(6) $$\frac{\rho_{f}\;}{\epsilon^{2}}\left(v.\nabla v\right)\;=\;-\nabla P\;+\;\mu_{eff}\left(\nabla^{2}v\right)\;-\frac{\mu_{f}}{K}v-\frac{\mu_{f}C_{F}}{\sqrt{K}}\left|v\right|v\;\;$$

Here, ϵ is the porosity of the porous medium (skin layers); *K* is the permeability; μ is the viscosity of the fluid flow in the microchannel; ∇P  is the pressure gradient; *C_F_* is the inertial coefficient; $\mu_{eff}$ is the effective viscosity and $\mu_{f}$ is the viscosity of the fluid flowing in the microchannel. Along with the DBF model, the mass conservation equation (i.e., Equation (5)) also needs to be taken into account while performing any simulations.

### 4.2. Heat Transfer Model for the Skin

**Figure 9:**
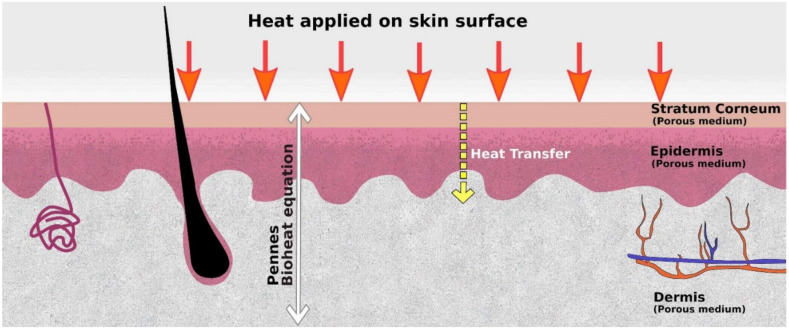
Representation of heat transfer across the dermal layers on the application of heat over the skin surface. The Pennes Bioheat transfer model is a basic model that represents the heat transfer in the skin.

Exposure of human skin to heat can impact the efficacy of topical/transdermal drugs. Understanding the effect on heat would shed light on the evaluation of topic drugs. When one applies heat over the skin surface (see Figure 9), it gets conducted into the stratum corneum, epidermis, dermis, and subcutaneous layer. Researchers widely use the Pennes model [122] to model heat transfer in the skin and the underlying tissues. The model is given by the equation that follows

(7) $$\rho C\frac{\partial T}{\partial t}=\;\nabla \cdot k\nabla T+\;\rho_{b}C_{b}W_{b}\left(T_{a}-T\right)+\;Q_{met}$$

Here, ρ  is the density of tissues; C is the specific heat; *T* is the temperature; *t* is the time; *k* is the thermal conductivity of the tissue; *ρ_b_*, *C_b_* and *W_b_* are the density, specific heat, and perfusion rate of blood. The Pennes model is the first bioheat equation that describes heat transfer in human tissue. This equation includes the effect of blood flow on tissue temperature on a continuum basis. The first, second, and third terms on the right-hand side represent heat conduction, arterial blood heating, and metabolic heat generation.

The Pennes model has been successfully applied for heat transfer in the skin by several researchers. Hao and co-workers [123] presented a comprehensive review of how heat affects drug delivery in the skin. The authors summarized the effect of heat on the following: (a) Dermal clearance; (b) transdermal drug release and delivery through the skin; (c) skin permeation; (d) transdermal and topical drug delivery systems under in-vivo and in-vitro conditions. Heat exposure on the skin can have a significant effect on the transdermal and topical delivery of drugs. Exposure to heat can result in increased release of drugs and increased skin perfusion. The pharmacological properties of the drugs can also affect drug delivery in the presence of heat. Hence, a thorough evaluation of topical/transdermal drug delivery under different thermal conditions is important while developing such drugs. Silva and co-workers [124] presented a detailed review of computational modelling of bioheat transfer in human skin. In their review work, they considered both heating and cooling of the skin. They listed and compared several modelling conditions used in bioheat transfer studies performed by other researchers. They mentioned that Pennes’ equation is one of the most used ones for modelling skin heat transfer along with the Henriques and Moritz function, which is used for thermal damage.

In experimental works dealing with microfluidic skin-on-a-chip platform, if one wishes to know the effect of applied heat on the transdermal delivery of drugs in the skin layers lying in the microchannel, with a top layer exposed to heat, the Pennes model would be useful.

## 5. Summary and Conclusions

This article gives an overview of the state of the art of utilization opportunities for skin-on-a-chip technology and devices with different biological and engineering backgrounds. First, the diffusion materials are summarized (membranes, excised human and animal skins, and reconstructed skin tissues), and further, various miniaturized platforms are presented. In the next session, the application possibilities are summarized, including drug and active ingredient diffusion studies, toxicological studies, pharmacological and wound healing experiments, and cosmetological investigations on skin repair, inflammation, ageing and, finally, shear stress.

The shear stress occurring during fluid flow in a microchannel aids in the formation of different layers of human skin (dermis, epidermis, and epithelium) with varied permeability over a porous membrane in a microfluidic platform, which can mimic the in-vivo conditions in a microenvironment; will provide nutrients and metabolites to the skin cells to keep them in an appropriate condition to test the drug.

Recent developments in microfluidic technologies show great potential in implementing a complex and in-vivo-like skin-on-a-chip model for drug testing. However, there are many challenges to be addressed: (1) One of the significant challenges is to mimic the structural complexity of living human skin, including vascularization (blood microvessels), immunity (dendritic cells, T-cells, macrophages, Langerhans cells), appendages (hair follicles, sweat glands, sebaceous glands, papillae) pigmentation (melanocytes), subcutis (adipocytes) and innervation (sensory afferents). (2) Control of the skin microenvironment, monitoring and analysis of drug effects in a user-friendly manner, which can be achieved by appropriate design and fabrication of microfluidic systems; (3) combining the technology with innovative detection methods (e.g., biosensors). Currently, bioengineered skin models are developed using animal cells or specific human cell types like keratinocytes and fibroblasts rather than various human skin cell types. Induced pluripotent stem cells (iPSCs) can almost infinitely differentiate into all skin cell types, which can solve this problem [125,126]. iPSC-derived skin cells can be used for developing personalized skin-on-a-chip models with relevant skin components (like immune cells and skin appendages) and possess barrier and dermal properties.

Overall, the development of next-generation skin-on-a-chip microfluidic platforms moves closer to testing with real, human skin and will help replace the animal models. The physical phenomena of drug diffusion and heat transfer into the dermal layers can be predicted through mathematical modelling and CFD simulations. CFD can accurately explain the diffusion of drugs of any new drug formulation into the dermal layers, which is usually not possible in in-vivo experiments or ex-vivo experimental studies. The porosity and permeability vary across the dermal layers. These parameters can be taken into consideration quickly while performing numerical simulations. It can also complement the studies carried out on microfluidic skin-on-a-chip platforms. It will reduce the cost of the development of new cosmetics and drugs related to skincare.

The skin-on-a-chip devices can be parallelized and can probably be connected to robotic systems which make it possible to perform medium-through-put screening or drug testing in the pharmaceutical or cosmetic industry. However, currently this technology is under the phase of validation and optimization with fabrication of prototype chambers and individual microfluidic devices. On the other hand this technology can later be routinely used in the early phase of drug development and cosmetic testing using reconstructed humans skin models or artificial skin equivalents. To generate a reliable skin-on-a-chip model, bioengineers, biologists, pharmacologists, and biostatisticians should collaborate. Their joint work on designing, fabricating, refining, and validating a skin-on-a-chip model will reduce animal experimentation and accelerate drug research.

## Figures and Tables

**Table 1:** Membranes used in diffusion studies as a surrogate of excised skins [9].

Membranes	Materials	Pros	Cons
Silicon based	Silastic, Polydimethylsiloxane (PDMS), Silatos	Cost effective, good storage conditions, good reproducibility, low variability	Fails to incorporate components like metabolism, distribution, and excretion
Cellulose-based	Pure cellulose, Cellulose-acetate, cellulose nitrate (glycerin and preservatives can be added for better flexibility)	Cost effective, good storage conditions, good reproducibility, low variability, very low protein binding capacity, hydrophilic, improved solvent resistance	Fails to incorporate components like metabolism, distribution, and excretion, lubrication is needed, not lipophilic
Synthetic polymer based	Nylon (aliphatic polyamides) (hydrophobic), polysulfone, polycarbonates (high flux membranes)	Low protein binding, chemical inertness, cost-effective, lack of tortuosity of the pores, good chemical stability	Higher cost, lower availability, fails to incorporate components like metabolism, distribution, and excretion
Strat-M^®^	Multilayer polyester sulfone polyolefin	Multiple layers with different permeability good storage conditions good reproducibility low variability, good correlation with excised skin	Fails to incorporate components like metabolism, distribution, and excretion
Chitosan	Chitosan-alginate Poloxamer 188	Porosity can be varied based on molecular weight and origin (fungal or animal) good physicochemical properties, thickness, roughness, opacity, liquid uptake, and water vapor permeability can be modified, non-toxic	Fails to incorporate components like metabolism, distribution, and excretion

**Table 2:** Characteristics of skins of different species (modified from Liu et al., 2009 [32]).

	Mouse	Rat	Porcine	Human
Skin thickness	0.4–1 mm	1–2 mm	1.5–2 mm	2–3 mm
Epidermal thickness	9.4–13.3 µm	21.7 µm	52–100 µm	50–100 µm
Stratum corneum	2.9 µm	5 µm	12.28 µm	10–12.5 µm
Fixed skin	no	no	Yes	Yes
Hair follicles	658 hairs/cm^2^	289 hairs/cm^2^	11 hairs/cm^2^	11 hairs/cm^2^
Sources	laboratory animals	laboratory animals	veterinary education, food industry	cadaver, tissue bank, biopsy

**Table 3:** These skin models are commercially available [35,36,37]. They are mostly used in the areas such as skin irritancy corrosivity testing, phototoxicity, tissue replacement in burns and bruises, and transdermal permeation studies.

Reconstructed Human Epidermis Models (RHE)		Full-Thickness Human Skin Models (LSE)	
EpiDerm	MatTek Corporation, Ashland, MA, USA	EpiDermFT	MatTek Corporation, Ashland, MA, USA
EpiSkin	L’Oréal, Lyon, France	StrataTest	Stratatech, Madison, WI, USA
reconstructed human epidermis	SkinEthic, Lyon, France	Phenion Full-Thick-ness Skin	Phenion, Düsseldorf, Germany
EpiCs	CellSystems, Troisdorf, Germany	GraftSkin	Apligraf; Organogenesis, MI, USA
open source reconstructed epidermis model	Phenion, Düsseldorf, Germany	Vitrolife-Skin	Kyoto, Japan
Straticell	Straticell, Les Isnes, Belgium		
Labcyte	Gamagori, Japan		

**Table 4:** An overview of tissue-engineered 3D skin models from human primary cells and their limitations (from Broek et al., 2017 [38]).

Model	Commercially Available	Advantages/Disadvantages	Ref.
Reconstructed epidermis	Yes: EpiDerm™, EpiSkin™, SkinEthic™, epiCS^®^ No: in house models	+: differentiated epidermis from keratinocytes −: only keratinocytes, no dermal compartment present, or immune cells	[39,40]
Pigmented Reconstructed epidermis	Yes: MelanoDerm No: in house models	+: pigmented differentiated epidermis from keratinocytes and melanocytes −: no living dermal compartment, immune cells, adipose tissue, appendages, or blood vessels present	[41,42]
Full-thickness skin models	Yes: EpiDerm-FT, Phenion-FT, LabSkin No: in house models	+: differentiated epidermis on the fibroblast-populated dermis −: no immune cells, adipose tissue, appendages, or blood vessels	[43,44,45,46]
Three-layered skin model	No: in house models	+: differentiated epidermis on fibroblast-populated dermis on an adipocyte/ASC populated hypodermis −: no immune cells or appendages	[47,48,49]
Full-thickness skin model containing EC	No: in house models	+: differentiated epidermis on fibroblast and endothelial cell (show vessel-like structures) populated dermis −: no immune cells, adipose tissue, appendages, or perfused blood vessels	[50,51]
Skin equivalent with integrated Langerhans Cells	No: in house model	+: pigmented skin model containing functional MUTZ-3 derived Langerhans −: no adipose tissue, appendages, or blood vessels	[52,53]

**Table 5:** Comparison of properties of traditional diffusion cell systems and skin-on-a-chip microfluidic devices.

Traditional Diffusion Devices	Skin-On-A-Chip Devices
high tissue need	low tissue need
high active ingredient need	low active ingredient need
high formulation need	low formulation need
macroscale size	microscale size
static system	dynamic system
poor reproducibility	good reproducibility
only ex vivo (or in vitro membranes)	ex vivo and in vitro membranes or cell cultures
high sample volumes	low sample volumes
high cost	low cost
controlled parameters	precisely controlled parameters

**Table 6:** Characteristics of different skin-on-a-chip systems in the literature (modified from Sutterby and co-workers, 2020 [65]). PDMS: Poly(dimethylsiloxane), ECM: Extracellular matrix, PET: Polyester, PMMA: Polymethylmethacrylate, PCL: Polycaprolactone, dECM: Dermal extracellular matrix.

Materials of the Chip	Fabrication Technology	Testing Features	Reference
PDMS	lithography	toxicity testing, high throughput	[66]
PDMS, PDMS membrane, natural ECM	lithography	multiorgan chip optimization of the parameters	[67]
PDMS, natural ECM	lithography	efficacy testing ex vivo using skin micro-biopsy	[68]
PDMS, collagen ECM	lithography	skin wrinkling cosmetic testing	[69]
PDMS, PET membrane, collagen ECM	lithography	drug testing pump free system	[59]
PDMS, collagen ECM	lithography	multiple collagen sources were compared toxicity testing	[70]
PDMS, collagen ECM	lithography	ex vivo skin and hair, validation study	[60]
PDMS, PET membrane, fibronectin ECM	lithography	edema and inflammation	[71]
PDMS, fibrin with collagen	lithography	skin irritation	[72]
PDMS, polycarbonate membrane, collagen ECM	lithography	pump free system, multicell skin model	[64]
PMMA, polycarbonate membrane, fibrin ECM	CNC micro milling	micro-milling	[73]
PDMS, PET membrane	Laser cutting	three parallels, diffusion study	[74]
PDMS, PMMA, PET membrane	Laser cutting	three parallels and TEER sensor integrated, immune study	[75]
silicon rubber, collagen ECM	3D printing	blood vessels, diffusion study	[76,77]
PCL, skin-derived dECM	3D printing	fabricated with vascular channels, validation study	[78]

**Table 7:** Effect of associated shear stress on the skin cells in a microfluidic chip.

	Undisturbed Laminar Flow 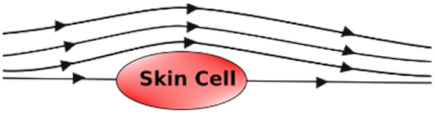	Disturbed Laminar Flow 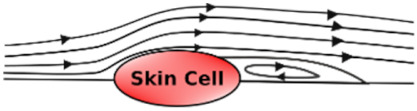	Turbulent Flow 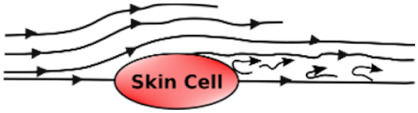
Porosity	Controlled porosity [100]	Mixed porosity	-
Permeability	Decreases [101]	Low	-
Wound Repair	Healing speed increases [102]	Healing speed is low	Healing speed is very low
Turnover rate	Low	High [103]	Very high
Inflammation	Very low [104]	High	Very high
Toxicology studies	Good toxicity results compared to static conditions [105,106]	-	-
